# The role of body size and cuticular hydrocarbons in the desiccation resistance of invasive Argentine ants (*Linepithema humile*)

**DOI:** 10.1242/jeb.245578

**Published:** 2023-08-21

**Authors:** Brian A. Whyte, Rebecca Sandidge, Jan Buellesbach, Elizabeth I. Cash, Kelsey J. Scheckel, Joshua D. Gibson, Neil D. Tsutsui

**Affiliations:** ^1^ Department of Environmental Science, Policy, and Management, 130 Mulford Hall, #3114, University of California, Berkeley, Berkeley, CA 94720-3114, USA; ^2^ Institute for Evolution and Biodiversity, University of Muenster, Hüfferstr. 1, D-48149 Münster, Germany; ^3^ Department of Biology, Georgia Southern University, PO Box 8042-1, Statesboro, GA 30460, USA

**Keywords:** Invasive species, Chemical ecology, Waterproofing, Communication, Supercolonies

## Abstract

An insect's cuticle is typically covered in a layer of wax prominently featuring various hydrocarbons involved in desiccation resistance and chemical communication. In Argentine ants (*Linepithema humile*), cuticular hydrocarbons (CHCs) communicate colony identity, but also provide waterproofing necessary to survive dry conditions. Theory suggests different CHC compound classes have functional trade-offs, such that selection for compounds used in communication would compromise waterproofing, and vice versa. We sampled sites of invasive *L. humile* populations from across California to test whether CHC differences between them can explain differences in their desiccation survival. We hypothesized that CHCs whose abundance was correlated with environmental factors would determine survival during desiccation, but our regression analysis did not support this hypothesis. Interestingly, we found the abundance of most CHCs had a negative correlation with survival, regardless of compound class. We suggest that the CHC differences between *L. humile* nests in California are insufficient to explain their differential survival against desiccation, and that body mass is a better predictor of desiccation survival at this scale of comparison.

## Teaser

**Highlighted Article:** Cuticular hydrocarbons help invasive ants survive desiccation and identify supercolony members. Some supercolonies have greater desiccation resistance, but this is likely due to differences in body size, not cuticular hydrocarbons.

## INTRODUCTION

The Argentine ant *Linepithema humile* is a globally widespread and damaging invasive species (Holway et al., 2002a) first introduced into North America in 1891 and established in California by 1907 (Suarez et al., 2001). In its introduced range, *L. humile* displaces native ant species, which can lead to cascading impacts on other organisms and the environment (Christian, 2001; Fisher et al., 2002; Holway et al., 2002a; Laakkonen et al., 2001; Sockman, 1997; Suarez and Case, 2002; Suarez et al., 2000). *Linepithema humile* are also serious structural pests (Klotz et al., 2008) and cause both direct and indirect agricultural damage (Holway et al., 2002a; Horton, 1918; Lach, 2005; Markin, 1970; Newell and Barber, 1913; Way, 1963). The invasive success of *L. humile* results, in large part, from the unusual colony structure of introduced populations (Tsutsui and Case, 2001; Tsutsui et al., 2000), which form geographically vast supercolonies (>1000 km in range) lacking territorial boundaries and dominating invaded ecosystems by sheer numerical superiority (Holway et al., 1998). In most introduced populations, widespread genetic homogeneity correlates with uniformity in the cuticular hydrocarbon (CHC) profiles that are used for nestmate recognition (Brandt et al., 2009a; van Wilgenburg et al., 2010), contributing to the expansive cooperative behavior within supercolonies (Brandt et al., 2009a; Torres et al., 2007). Although a single supercolony occupies nearly the entire introduced range in California, several small, genetically and chemically distinct colonies also occur in southern California (Suarez et al., 1999; Tsutsui and Case, 2001; Tsutsui et al., 2000).

In general, as major components of the waxy lipid layer secreted by the epicuticle of insects, CHCs constitute a fundamental barrier to protect against desiccation and microbial infections (Blomquist and Bagnères, 2010; Blomquist et al., 1987, 1998; Gibbs, 2002, 2011). Small-bodied animals, such as ants, have a high surface area to volume ratio, and this makes them particularly susceptible to desiccation (Kühsel et al., 2017). *Linepithema humile*, in particular, show lower survival rates in xeric habitats compared with other ant species (Holway et al., 2002b; Schilman et al., 2005). CHCs of *L. humile* are composed primarily of straight-chain *n*-alkanes, straight chain *n*-alkenes, as well as mono-, di- and tri-methyl-branched alkanes (Brandt et al., 2009b; Buellesbach et al., 2018), and each of these CHC classes possesses different physical characteristics that determine their efficacy in preventing water loss through the cuticle. Lower melting temperatures (*T*_m_), for example, correspond to higher cuticular permeability and lower efficacy in preventing desiccation (Blomquist and Ginzel, 2021; Gibbs, 2002, 2011). Within CHC classes, *T*_m_ increases with longer carbon chains (Blomquist and Ginzel, 2021). Across classes, for CHCs of equal carbon chain length, *n*-alkanes have 20–50°C higher *T*_m_ than CHCs that possess double bonds or methyl branches (Gibbs, 2002; Gibbs and Pomonis, 1995). Thus, selection for higher desiccation resistance in xeric habitats might drive the evolution of CHC profiles containing a larger proportion of long-chained *n*-alkanes and a lower proportion of *n*-alkenes and methyl-branched alkanes.

Invasive populations of *L. humile* are a fascinating model system for studying the functional trade-off between CHC classes, specifically *n*-alkanes and methyl-branched alkanes. Two functions of their CHC profile (nestmate recognition and desiccation resistance) are likely important in their success as a globally invasive species, but each function relies on different compound classes. Methyl-branched alkane variations appear to be predominantly responsible for nestmate recognition and mediating aggression between different supercolonies (Brandt et al., 2009b; Torres et al., 2007). *n*-Alkanes and *n*-alkenes, in contrast, appear to be more important for functional recruitment in desiccation prevention (Buellesbach et al., 2018).

In other ant genera, including *Pogonomyrmex*, *Temnothorax* and *Myrmica*, more desiccation-resistant profiles with proportionally higher *n*-alkane quantities and lower methyl-branched alkane quantities have been observed in more xeric conditions (Menzel et al., 2018; Sprenger et al., 2018; Wagner et al., 2001). A similar trend has also been observed in *L. humile*, where some methyl-branched alkanes correlated positively with precipitation rates and negatively with temperature, while other *n*-alkanes and *n*-alkenes correlated negatively with precipitation rates and positively with temperature at the respective nest habitats (Buellesbach et al., 2018). This raises an interesting question: do the invasive populations of *L. humile* in California, whose profiles communicate supercolony identity, also demonstrate acclimation or optimization for desiccation resistance? If so, this may contribute to differential invasion success between supercolonies.

To begin answering questions like this, we must first test whether there is a connection between these CHC compound classes and desiccation resistance in *L. humile*. We did this by collecting *L. humile* nests from multiple supercolonies from across California, recording 72 individual CHCs from their colony profiles, and assaying worker survival under experimental desiccation. We expected the quantities of CHCs closely associated with habitat temperature and precipitation (Buellesbach et al., 2018) to correlate with survival under increased desiccation stress. Moreover, we also considered how variation in body size influences survival under desiccation stress, as smaller body sizes generally translate to higher surface area to volume ratios, and thus higher susceptibility to desiccation (Kühsel et al., 2017).

## MATERIALS AND METHODS

### Ant nest collection

**Fig. 1.**
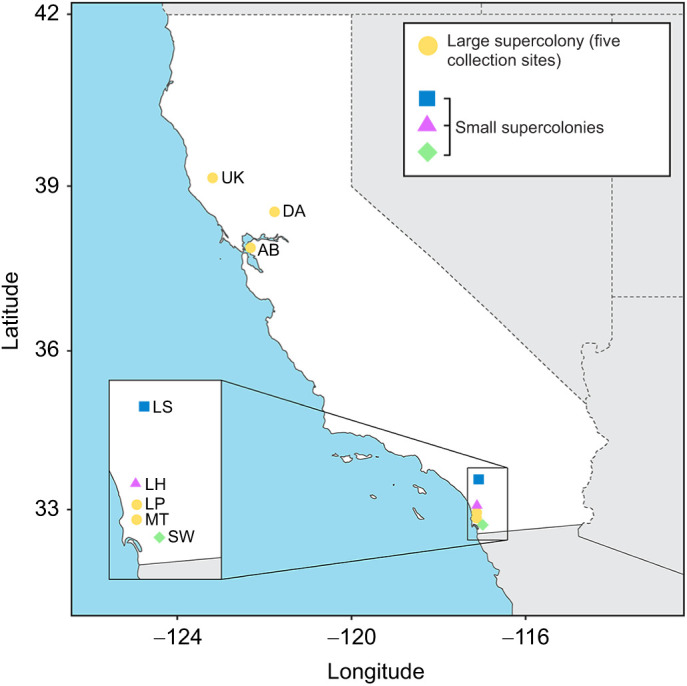
**Map of sampled *Linepithema humile* nest locations.** Letter abbreviations indicate collection site locations in California; symbol color and shape indicate supercolony identity. Large supercolony: UK, Ukiah; DA, Davis, AB, Albany Bulb; LP, Los Peñasquitos; MT, Mission Trails. Small supercolony: LS, Lake Skinner; LH, Lake Hodges; SW, Sweetwater.

We collected *Linepithema humile* (Mayr) nests from eight sites across California during January–May 2017 (Fig. 1). Five of these sites (Ukiah, Davis, Albany Bulb, Los Peñasquitos and Mission Trails) are members of the ‘large’ supercolony that dominates nearly all of their introduced range (Buellesbach et al., 2018; Tsutsui et al., 2003). We also collected nests from three of the separate ‘small’ supercolonies in southern California (Lake Skinner, Lake Hodges and Sweetwater) (Buellesbach et al., 2018; Tsutsui et al., 2003). Colony fragments were collected by excavating substrate (soil, leaf litter and/or decomposing wood) with a shovel or trowel and placing it into a ∼20 l plastic bucket. When the substrate was sandy or dusty soil, crumpled paper towels were added to provide structure and air gaps. In the laboratory, the collections were distributed in a ∼5–10 cm deep layer across the bottom of large (58.4 cm×41.3 cm×15.2 cm) plastic tubs. The ants were then provided with food (Bhatkar and Whitcomb, 1970) and water, and left undisturbed for 24-48 h to allow workers to collect scattered brood and consolidate colony members into cavities. The colony fragments were extracted from the substrate over the course of several hours by slowly flooding the tubs with water and providing a narrow paper bridge for relocation into an adjacent tub. The ants were contained in the new tubs by applying Insect-A-Slip (BioQuip Products, Inc., Compton, CA, USA) along the inner walls and placing the tub on bricks in a second tub that functioned as a moat of soapy water. Within the primary tubs were nesting containers, consisting of Petri dishes with a layer of plaster of Paris on the bottom, a small entrance hole drilled in the side, and a lid to block light. The ants were maintained at room temperature and humidity in the lab and used for desiccation resistance assays during their second week after collection.

### Desiccation resistance assays

**Fig. 2.**
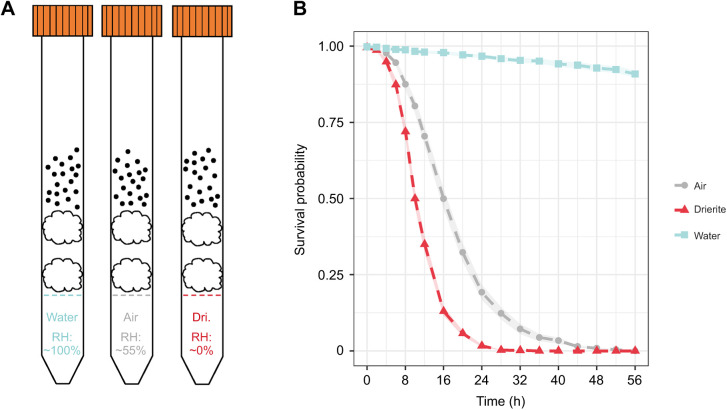
**Desiccation assay design and survivorship.** (A) Illustration of the 15 ml conical tubes used for the three humidity treatments: the ants (black dots) rest on top of cotton balls to separate them from the contents underneath, which influence the relative humidity (RH) of each tube. Left to right: water (RH=1, ‘negligible desiccation’), air (RH=0.55, ‘moderate desiccation’) and Drierite (RH=0, ‘severe desiccation’). (B) Survival probability of ants over time was combined over all assayed ants per treatment to show general survival patterns in each treatment. Standard errors of the mean are indicated in narrow ribbons around each survival curve.

Ant survival was quantified under three humidity treatments to impose different levels of desiccation stress: negligible desiccation [‘water’, relative humidity (RH)≈100%], moderate desiccation (‘air’, RH≈55%) and severe desiccation (‘Drierite’, RH≈0%) (Fig. 2A). Preliminary experiments using iButtons (iButtonLink^©^, Innovation Drive Whitewater, WI, USA) confirmed these RH estimates in each humidity treatment (Fig. S1). Water tubes were constructed by filling 15 ml Corning^®^ conical vials with ∼2 ml distilled water and pushing a cotton ball down to the top of the water (Fig. 2A). A second cotton ball was inserted above, creating an air gap of ∼4 mm between the two cotton balls. This acted as a barrier to prevent the ants from accessing drinking water or excavating into the water and flooding the tube. The remaining empty volume (∼7–8 ml) housed the worker ants during the desiccation assay. Air and Drierite tubes were constructed in the same manner, except air tubes had no water beneath the cotton balls, and Drierite tubes had ∼2 ml of desiccant (Drierite Co. Ltd., Xenia, OH, USA) at the bottom instead of water. All tubes were prepared the day before initiation of the desiccation assays, to allow RH to stabilize before the ants were introduced.

To perform desiccation resistance assays, we used aspirators to arbitrarily select 23 worker ants from the eight collected nests and placed them in small, temporary holding dishes [Petri dishes (35 mm diameter, 10 mm height) with a small cotton ball (7 mm) saturated with 200 µl of water] for 24 h before initiation of desiccation assays. This was repeated 10 times for each of the three humidity treatments for each colony, leading to a total of 240 assay tubes. Only 20 ants from each replicate were transferred to their respective assay tube as the extra three ants were to account for possible deaths or escape in the temporary holding dishes. Occasional miscounts led to instances of 19 (*n*=4) and 21 (*n*=1) ants occurring out of the total 240 assay tubes. We recorded the mortality of ants in each tube every 2 h for the first 12 h, then every 4 h until there were no surviving ants in the air and Drierite tubes (water tubes experienced little to no mortality during the assay). We assessed mortality by lightly tapping the sides of the tubes and rolling them horizontally to observe the ants. All ants that were moving, even if not standing, were counted as alive. Ants that did not move during this assessment were scored as dead.

Only ants from the Drierite tubes were used for body mass measurement after all ants had died, because they were less likely to be degraded by moisture. Samples were stored at −20°C immediately after the desiccation assays and remained frozen until processing. For measuring body mass, desiccated worker ants from the Drierite tubes were removed, counted and placed in clean 2 ml microcentrifuge tubes with snap tops. These tubes, with lids open, were placed in an airtight container on a 4 cm layer of Drierite for 3 days at room temperature. The dry mass of each sample was weighed, and divided by the number of individual ants in the sample to provide an average mass for the ants of each sample tube.

### GC-MS analysis of CHCs

CHCs were extracted and analyzed by gas chromatography coupled with mass spectrometry (GC-MS) as described previously (Buellesbach et al., 2018). We used workers collected directly from the same nests used in our desiccation assays to determine representative CHC profiles for each nest.

Individual workers were placed into 2 ml screw-top GC vials (Agilent Technologies, Santa Clara, CA, USA), 100 μl of hexane (HPLC grade, Fisher Scientific, Fair Lawn, NJ, USA) was added, and the vials were swirled for 10 min on a Thermolyne Roto Mix (Marshall Scientific, Hampton, NH, USA). These extracts were transferred to a conical 250 μl GC insert (Agilent Technologies), and subsequently evaporated under a flow of nitrogen gas (Praxair, Inc., Danbury, CT, USA). The dried extract was then resuspended in 10 μl hexane with 7.5 ng μl^−1^ *n*-dodecane (EMD Millipore Corp., Billerica, MA, USA) as an internal standard.

Half of the resuspended CHC extract (5 μl) was injected into a gas chromatograph coupled with a mass-selective detector (GC: 7890A; MS: 5975C; Agilent Technologies), which was operated in electron impact ionization mode. The injection was performed in a split/splitless injector in the splitless mode with an inlet temperature of 250°C. Compounds were separated on a fused silica capillary column (J&W DB-5ms low-bleed GC columns, 30 m×0.32 mm×0.25 μm, Agilent) using a temperature program starting at 80°C for 5 min and increasing by 80°C min^−1^ to 200°C, followed by an increase of 5°C min^−1^ to 325°C, which was then held for 3 min. Helium was used as carrier gas at a constant flow rate of 1.8 ml min^−1^. Peak area integration and calculation were performed using G1701EA Enhanced ChemStation software v.E.02.02 (Agilent Technologies). Peaks were automatically integrated with an initial area reject of 0, an initial peak width of 0.017 and an initial threshold of 13, with shoulder detection turned off. CHC compounds were identified according to their retention indices, diagnostic ions and mass spectra. Absolute quantities (in ng) for a total of 72 individual CHC compounds across the eight collected nests were determined based on the internal *n*-dodecane standard. To control for the effect of larger ants having larger body surfaces requiring larger amounts of CHCs, we weighted our CHC absolute quantities (in ng) by surface area, estimated from body masses recorded from the Drierite treatment groups. Following Kühsel et al. (2017), we multiplied body mass by a hymenopteran body parameter (unitless) and calculated the cubed root of the squared body mass (mg^2/3^). Therefore, CHC mass is reported in ng mg^−2/3^ (CHC mass per surface area). We also calculated an alternative transformation of CHC abundance, standardized against a randomly chosen candidate CHC profile, to consider CHC abundance without being transformed by body size. This alternative CHC dataset will hereafter be referred to as the ‘relative CHC abundance’. These results are referenced in the Results, with figures and analyses reported in Figs S3 and S5, and Data 4 (the latter is available from Dryad at doi:10.6078/D15T50).

### Statistical analysis

All data were analyzed in the statistics programming language R (v.4.2.2, http://www.R-project.org/). To assess the effect of our humidity treatments, we compared survival curves of all ants from all colonies grouped by humidity treatment (Fig. 2B). As mortality was nearly absent in the water treatments, we only analyzed the air and Drierite groups (separately) when comparing nest survival rates. We measured survival of each nest by calculating median lethal time (LT50) for each sample tube (R command ‘dose.p’, R package ‘MASS’, https://CRAN.R-project.org/package=MASS), which measures the time at which half of the ants in each tube were predicted to be dead, and we averaged this for each nest (*n*=10 sample tube replicates). Significant differences between the nests were assessed with a pairwise permutations analysis of the variance (R command ‘pairwise.adonis’, R package ‘pairwiseAdonis’, https://github.com/pmartinezarbizu/pairwiseAdonis), using Euclidean distances, and significance levels were Benjamini–Hochberg corrected. Similarly, ANOVA tests were performed to test the differences between body size and CHC compound class between nests. The relationship between survival and body size (LT50 versus surface area) was analyzed with mixed effects regression models (R command ‘lmer’, R package ‘lme4’, https://CRAN.R-project.org/package=lme4).

We used a random forest regression model to perform feature selection on the 72 individual CHCs recorded from our nests, using LT50 in the desiccation trials as the response variable. We used the R package ‘Boruta’ (https://CRAN.R-project.org/package=Boruta; Kursa and Rudnicki, 2010), which categorizes the importance of our CHCs as ‘confirmed’, ‘tentative’ or ‘rejected’ based on their performance against ‘shadow’ attributes, generated from randomly sampling our CHC data. Boruta uses a *z*-score based measure of importance to account for mean accuracy loss across generated decision trees in the random forest (Kursa and Rudnicki, 2010). A feature that is categorized as ‘tentative’ or ‘rejected’ does not obtain significantly higher *z*-scores from the maximum *z*-score among shadow attributes, and we consider these features as uninformative for predicting desiccation survival (LT50). The data, code and output from the Boruta analysis can be found in Data 1, Code and Data 4 (available at doi:10.6078/D15T50).

## RESULTS

### Desiccation resistance varies by supercolony and nest

**Fig. 3.**
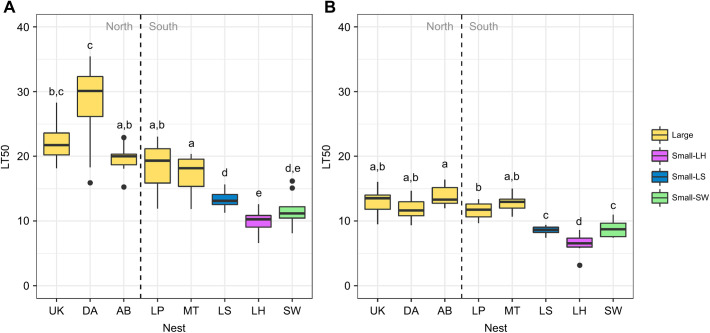
**Desiccation resistance of ants from each nest site.** Median lethal time (LT50) for each nest, separated for the air (moderate desiccation, RH≈0.55; A) and Drierite (severe desiccation, RH≈0; B) treatments. The vertical dashed lines separate the geographic regions (north/south California) of the nests (see Fig. 1 for more details). Letters indicate significance groups within (but not across) the two conditions, determined by a pairwise permutations ANOVA (full test results are given in Data 2 at doi:10.6078/D15T50).

Survival was high in the negligible desiccation treatment (water, RH=1), lower in the moderate desiccation treatment (air, RH=0.55) and lowest in the severe desiccation treatment (Drierite, RH=0) (Fig. 2B). While both the moderate and severe desiccation treatments reached 100% dead by 58 h, the negligible treatments on average only had 9% dead. Focusing on the moderate and severe treatments, we found significant variation among nests in their capacity to resist desiccation (Fig. 3; Data 1 at doi:10.6078/D15T50). In the moderate treatment, while some nests collected from the large supercolony (particularly Davis) had significantly different mean LT50 values from each other, all the collections sampled from sites belonging to the large supercolony had significantly higher LT50 values than those from the small supercolonies (Lake Skinner, Lake Hodges, Sweetwater). The performance of ants from nests that belong to the large supercolony were more similar to each other in the severe desiccation treatment (Fig. 3B), but all large supercolony nests still had significantly higher LT50 values than the small supercolony nests.

### Body size influences desiccation resistance and varies by supercolony

**Fig. 4.**
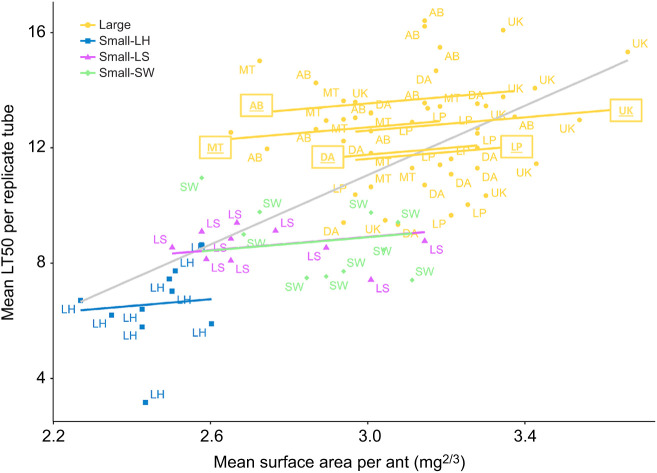
**Linear regression models.** Each point is labeled with nest ID, with symbol color and shape indicating supercolony identity (see Fig. 1). Body mass was only recorded from ants in the Drierite treatment group, so only LT50 data from that treatment were used. The gray line (*R*=0.61, *P*=2.7e−09) indicates the population mean of *y*≈*x*, while the colored lines indicate random intercepts for nest ID, determined from mixed effects models including random effects for nest and supercolony ID. The model information can be found in Data 2 and the Code (doi:10.6078/D15T50).

Body size (measured in mg^2/3^ to represent body surface area) was only collected from the ants used in the Drierite treatment (see Methods and Materials), so for testing the effect of body size on survival, we only considered the survival data (i.e. LT50) from the Drierite treatment. Body surface area had a significant, positive correlation versus LT50 (Fig. 4, gray line) with a slope of 6.018. However, a model allowing random effects for nest ID nested within supercolony ID yielded significantly lower AIC scores (ΔAIC=38.13, χ^2^=42.13, *P*=7.10e−10; see Data 2 at doi:10.6078/D15T50) and a slope of 1.154. Nests significantly differed in surface area, with ants from the large supercolony possessing the highest values (Fig. S2). On average, the largest ants were from Ukiah, and the smallest ants were from Lake Hodges (Fig. S2).

### CHC compound class differences between nests

**Fig. 5.**
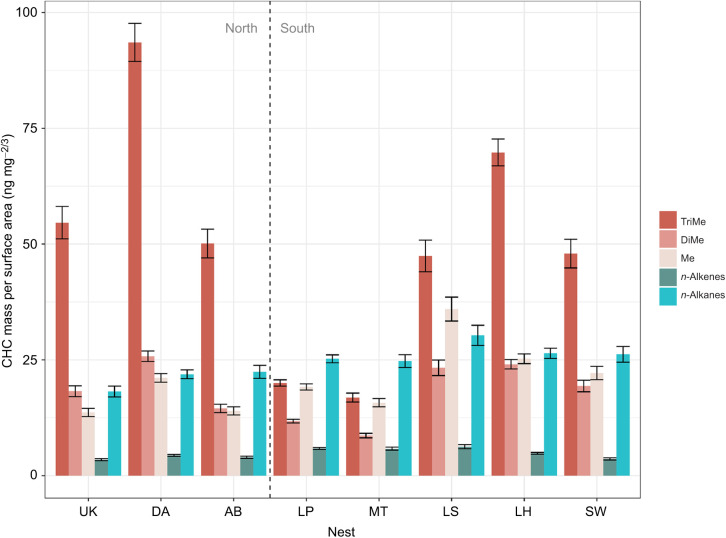
**Cuticular hydrocarbon class composition for each nest.** Each bar is the average cuticular hydrocarbon (CHC) mass per body surface area (ng mg^−2/3^) from the replicate groups used in the severe desiccation treatment (Drierite, RH=0). The five color classes are *n*-alkanes, *n*-alkenes, and methyl-branched alkanes with one (Me), two (DiMe) or three (TriMe) methyl branches. For nest abbreviations, see Fig. 1. The vertical dashed line separates nests collected in north or south California. Results from ANOVA tests of CHC class differences within and between nests can be found in Data 2 at doi:10.6078/D15T50.

Of the 72 CHCs extracted from our nests, three were *n*-alkenes, 14 were *n*-alkanes, 19 were mono-methyl alkanes, 17 were di-methyl alkanes and 19 were tri-methyl alkanes. All identified CHCs, along with relevant information such as retention index and estimated methyl group locations, can be found in Data 3 (doi:10.6078/D15T50). A multivariate analysis of variance (MANOVA) revealed significant differences in CHC class abundance across nests (Data 2 at doi:10.6078/D15T50). For all nests except Mission Trails and Los Peñasquitos, tri-methyl alkanes had the highest absolute abundance of all compound classes recorded in their CHC profile (Fig. 5, Fig. S4). The nest with the largest mass of tri-methyl alkanes was Davis, from the large supercolony, but the nest with the second largest mass of tri-methyl alkanes was Lake Hodges, which does not belong to the large supercolony (Fig. 5, Fig. S4). However, when using relative CHC abundance instead of CHC mass weighted by surface area, the above patterns did not hold (Fig. S3). Tri-methyl alkanes only remained as the most abundant class for the northern large supercolony nests (Ukiah, Davis and Albany Bulb), while all other nests possessed predominantly *n*-alkanes, except for Lake Skinner, where mono-methyl alkanes were the predominant CHC class.

### Random forest feature selection of individual CHCs

**Fig. 6.**
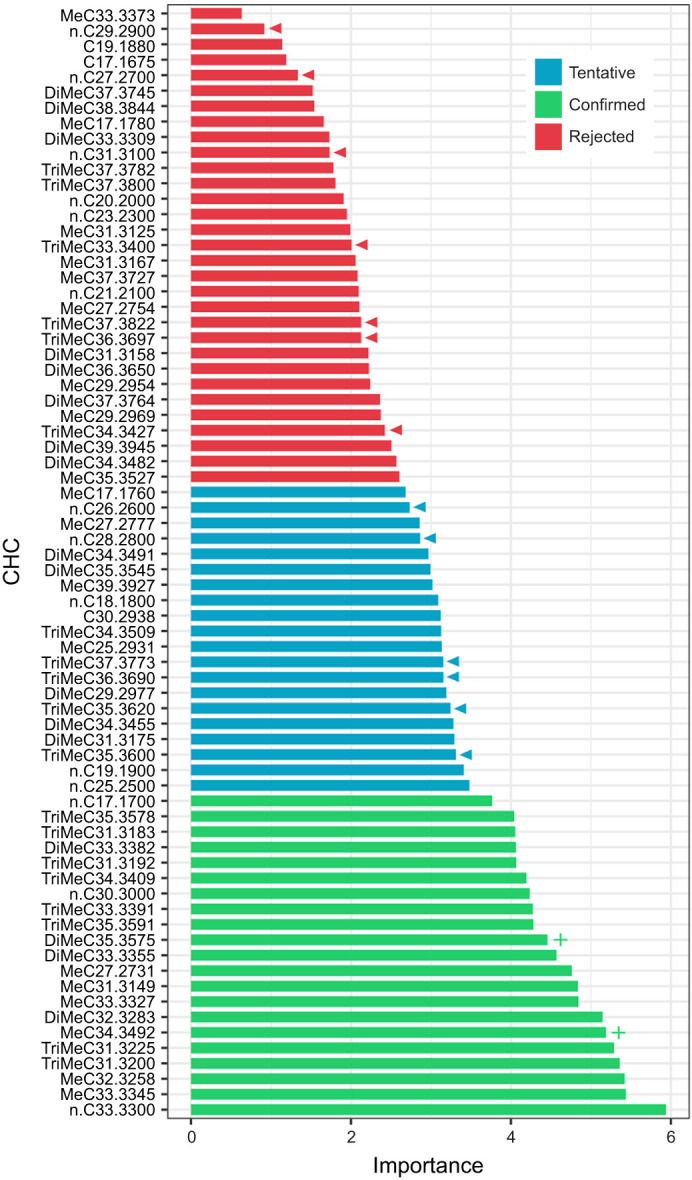
**Random forest feature selection.** The features are all of the recorded CHCs found from our nest collections, used in random forest decision trees to predict LT50 (from the Drierite treatment, specifically). The abbreviations of each CHC on the *y*-axis refer to the compound class (*n*, *n*-alkane; Me, mono-methyl alkane; DiMe, di-methyl alkane, TriMe, tri-methyl alkane; no prefix, *n*-alkene), carbon chain length (C followed by two digits) and retention index (last four digits). Decision categories were determined by the Boruta feature selection in R (Kursa and Rudnicki, 2010). Arrowheads indicate CHCs that were found to be significantly correlated to climate conditions in our previous study (Data 3 at doi:10.6078/D15T50; Buellesbach et al., 2018). Within the green ‘confirmed’ CHCs, the ‘+’ indicates the only CHCs that are positively correlated with LT50. Spearman correlation results for each CHC can be found in Data 3.

Our random forest feature selection using the Boruta package in R (Kursa and Rudnicki, 2010) found that, out of the 72 individual CHCs identified on our ants, 21 of them (29.1%) were selected as significant features for predicting LT50 in decision trees (i.e. ‘confirmed’, Fig. 6). Of these top 21 CHCs, three were *n*-alkanes (14.3%), six were mono-methyl alkanes (28.6%), four were di-methyl alkanes (19%) and eight were tri-methyl alkanes (38.1%). All of these selected CHCs, regardless of compound class, were significantly negatively correlated with LT50 from the Drierite group except for two: 5,15- and 5,17-DiMeC35 (retention index: 3575) and 12-, 14- and 16-MeC34 (retention index: 3492), which were significantly positively correlated with LT50 (Fig. 6, plus signs). The Spearman coefficients and Benjamini–Hochberg corrected *P*-values for all CHCs (including those not selected by Boruta) can be found in Data 4 (doi:10.6078/D15T50). The CHCs found to be significantly correlated with climate conditions in our past study (Data 4; Buellesbach et al., 2018) were all considered to be rejected or tentative in the Boruta feature selection (Fig. 6, arrowheads). Similarly, none of the specific CHCs that were previously identified by Brandt et al. (2009a,b) as colony recognition cues were found to be associated with desiccation resistance in our random forest analysis (15-MeC35 and 17-MeC35, shown here as MeC35.3527; 5,13,17-TriMeC35, shown here as TriMeC35.3600; 5,13,17-TriMeC37, shown here as TriMeC37.3800).

When using relative CHC abundance in a Boruta feature selection instead of CHC mass weighted by surface area, the overall result is the same. This alternative Boruta analysis (Fig. S5; Data 4 at doi:10.6078/D15T50) found only 18 ‘confirmed’ CHCs instead of 21, but all 18 of those CHCs were found in the confirmed category of the primary Boruta analysis (Fig. 6). Of these 18 confirmed CHCs, their correlations versus LT50 were the same, with only 5,15-, 5,17-DiMeC35 (retention index: 3575) and 12-, 14-, 16-MeC34 (retention index: 3492) being significantly positively correlated with LT50 (Fig. S5, plus signs). Additionally, all CHCs found to be significantly correlated with climate conditions in our past study were rejected in this alternative Boruta as well (Fig. S5, arrowheads).

## DISCUSSION

The ability of an organism to resist desiccation can be influenced by a myriad of factors, including physical characteristics of the organism, its life history and ontogeny, and potential behavioral responses (Bujan et al., 2016; Thorat and Nath, 2018; Wang et al., 2021). In this study, we quantified desiccation resistance in a desiccation-sensitive invasive species and examined several potential drivers of desiccation sensitivity. We found consistent differences among introduced *L. humile* supercolonies in their ability to resist desiccation. Because the largest supercolony in California is geographically widespread (∼1000 km long), comparisons of populations from distant sites provide insights into how these genetically similar ants, all with the same supercolony identity, perform in different environments. Additionally, comparison with the geographically restricted small supercolonies provides insight into how desiccation resistance varies both across and within supercolonies.

In both moderate (RH=50%) and severe (RH=0%) desiccation treatments, ants from the large supercolony survived the longest, having significantly higher LT50 values (Fig. 3). Colony structure and displacement of native ant species have been linked to the success of this supercolony in its introduced range (Holway et al., 1998; Tsutsui and Case, 2001; Tsutsui et al., 2000). Our data suggest that heightened ability to resist desiccation relative to other *L. humile* supercolonies may also contribute to its success by conferring greater ability to persist in xeric habitats, such as the Mediterranean climate of coastal California.

However, it remains unclear how these differences in desiccation resistance among introduced supercolonies affect interspecific competition. Previous research has shown that body size accounts for much of the difference in water loss rates across several different ant species (Schilman et al., 2007). Moreover, when compared with native ants in southern California, *L. humile* generally has higher cuticular permeability and is significantly more susceptible to water loss and lethal desiccation (Holway et al., 2002b; Schilman et al., 2007). These physiological characteristics may still prevent *L. humile* from invading habitats with low humidity. Irrigation experiments in the field, for example, have shown that water subsidies allow *L. humile* to spread into formerly inhospitable arid habitats and, conversely, cessation of irrigation leads to their withdrawal (Menke and Holway, 2006; Menke et al., 2007).

Under moderate desiccation stress, we found more variation in performance than was evident under severe desiccation stress, especially within the large supercolony (Fig. 3A). In this treatment, large supercolony ants from Davis were characterized by significantly higher levels of desiccation resistance than all others except Ukiah, and ants from Mission Trails showed significantly lower desiccation resistance than ants from Ukiah and Davis (while not different from those from Albany Bulb or Los Peñasquitos). The causes of this variation are unclear but may indicate seasonal or environmental differences among these sites that are only manifested under low levels of desiccation stress. Indeed, because the humidity levels in this treatment were not particularly dry (RH≈50%; Fig. S1), the differential mortality rates observed in this treatment may have arisen from drivers other than desiccation.

Patterns were clearer under severe desiccation stress (RH=0%). All ants from sites within the large supercolony exhibited nearly identical levels of desiccation resistance (Fig. 3B), despite being collected from sites separated by as much as 900 km (Mission Trails to Ukiah). The large difference in latitude among our collection sites translates into a variety of environmental differences. For example, focusing on the most distant sites sampled from the large supercolony, the southern Mission Trails site receives only about 32.3% of the average annual rainfall compared with the northern Ukiah site (http://www.worldclim.org; Fick and Hijmans, 2017). The similarity in desiccation resistance exhibited across all these sites within the large supercolony, despite their heterogeneous environments, suggests a genetic basis for desiccation resistance, and a limited role for phenotypic plasticity.

Multiple factors potentially contribute to the superior desiccation resistance observed in the large supercolony (Fig. 4), and we hypothesized that CHC profiles are one of these factors. Within the large supercolony, the southern nests (Los Peñasquitos and Mission Trails) have relatively low amounts of tri-methyl alkanes, and relatively high amounts of *n*-alkanes in their profile (Fig. 5) compared with their northern supercolony counterparts (Ukiah, Davis, Albany Bulb). Our alternative analyses of CHC classes, using relative abundance instead of CHC mass per surface area, also found the same pattern, where northern nests had relatively higher amounts of tri-methyl alkanes (Fig. S5). This agrees with acclimation patterns seen in other ant species (Menzel et al., 2018; Sprenger et al., 2018), though this difference was not upheld within the smaller supercolonies, as their *n*-alkane proportions were lower than those of both Los Peñasquitos and Mission Trails. In a previous study (Buellesbach et al., 2018), we found correlative evidence of profile acclimation, where the abundance of some *n*-alkanes negatively correlated with precipitation (while also positively correlating to temperature), and that of some methyl-branched alkanes positively correlated with precipitation (while also negatively correlating with temperature) (Data 4 at doi:10.6078/D15T50).

Surprisingly, none of these specific *n*-alkanes or methyl-branched alkanes were selected features in our random forest regression (Fig. 6, arrowheads), suggesting that these compounds that correlate in abundance with climate conditions (i.e. precipitation and temperature; Buellesbach et al., 2018) may not be useful for predicting survival under severe desiccation stress (RH≈0). Every compound class except *n*-alkenes was represented in the selected features (Fig. 6, ‘confirmed’ CHCs), but the majority of these compounds (90%) were significantly negatively correlated with survival. Having the majority of the feature-selected CHCs, regardless of compound class, negatively correlate with survival is unexpected.

This surprising result, which does not confirm physiological considerations on CHC profile compositions best suited for waterproofing (Blomquist and Bagnères, 2010; Blomquist et al., 1987, 1998; Gibbs, 2002, 2011) could potentially be explained by multiple confounding factors. First, our experimental design differed from similar studies (Menzel et al., 2018; Sprenger et al., 2018). Those studies have shown that ants acclimated to low humidity levels for 3 weeks have increased survival under conditions of high desiccation stress, which was correlated with increased proportions of *n*-alkanes. Our experiments followed a different protocol, acclimating the ants for 1 week under neutral conditions (21–26°C, ∼50% RH) before the experiment, which perhaps reversed or weakened the acclimation of the ant populations from their respective habitats. Though not a confounding variable, the northern nests (Ukiah, Davis, Albany Bulb), which are all in environments with higher precipitation rates than the southern nests (Los Peñasquitos, Mission Trails, Lake Skinner, Lake Hodges, Sweetwater) (Fick and Hijmans, 2017), unexpectedly showed the highest survival rates against desiccation stress (Fig. 3). This challenges the hypothesis that ants from more xeric habitats would display higher resistance against desiccation than ants less acclimated to these conditions.

We also hypothesize that the scale of our analysis – between nests of the same species – generally displays a low level of quantitative hydrocarbon variation between our study populations, as well as few qualitative CHC differences, such that the expected effect of CHC abundance on survival seen in other studies (Baumgart et al., 2022; Ferveur et al., 2018; Sprenger et al., 2018; Wang et al., 2022) was absent in our results. Of the 72 CHCs identified from our nests, only nine were not universal (i.e. not found in every nest profile) and, coincidentally, all nine of these were in the 21 feature-selected CHCs (Fig. 6; ‘confirmed’). All of these non-universal CHCs were long-chained (C31-35) di- and tri-methyl alkanes. While this may suggest that presence/absence of these large methyl-branched hydrocarbons drives desiccation resistance, we hesitate to support this interpretation. Many other di- and tri-methyl alkanes with similar chain lengths were rejected in our feature selection, and we have no evidence to suggest that compounds with such similar chemical properties (compound class and chain length range) would have different waterproofing properties.

Typically, methyl-branched alkanes have lower melting points than *n*-alkanes of equivalent carbon chain length, and exhibit greater cuticular permeability (Blomquist and Ginzel, 2021; Gibbs, 2011; Gibbs and Pomonis, 1995), which should translate into greater compromising of desiccation resistance. Nonetheless, some case studies have indeed shown greater waterproofing and desiccation resistance in insects with long-chain methyl-branched alkanes. A study on invasive *Solenopsis* species, using differential scanning calorimetry to measure melting points of entire CHC profiles, found that profiles with increased methyl-branched alkane proportions exhibited higher overall melting points (Xu et al., 2018). Another study, also using random forest algorithms, found four methyl-branched CHCs (26–30 chain length) as the best predictors of *Drosophila* desiccation resistance across species (Wang et al., 2022). Wang et al. (2022) also showed that adding synthesized versions of these methyl-branched alkanes could significantly improve desiccation resistance in assays, but this varied by sex, and not all compounds had a significant effect. Notably, these two studies compared multiple species of ants or flies against each other, not multiple subpopulations of the same species, as in our study.

We know that CHCs are not the only factors driving survival under desiccation (Thorat and Nath, 2018), and our results show that body mass shares a significant positive correlation with survival (LT50) (Fig. 4). The ants with the highest average body mass were found in nests from the large supercolony (Ukiah, Davis, Albany Bulb, Los Peñasquitos), whereas the ants with the lowest average body mass were from one of the small supercolonies (Lake Hodges) (Fig. S2). This is consistent with their desiccation performance, as a larger body mass translates into a smaller surface area to body volume ratio (Kühsel et al., 2017), which is predicted to reduce water loss through the cuticle. This body mass difference between northern and southern nests of the large supercolony could contribute to the unexpected pattern of northern ants (hypothetically acclimated to relatively humid conditions) performing the best under desiccation stress.

Conversely, it is possible that the invasive success of the large supercolony translates into increased body size of workers, thus enhancing desiccation resistance. For example, within the large geographic areas dominated by the large supercolony, *L. humile* can monopolize food and nesting resources, while also establishing long-term mutualisms with phloem-feeding Homoptera (Holway et al., 2002a). The resulting large amount of nutritional resources that flow into nests of the large supercolony may allow developing workers to attain larger adult body sizes which, as we show here, confer heightened desiccation resistance. If such a causal linkage occurs between invasive success, body size and desiccation resistance, this could create a positive feedback loop in which invasive success is further increased, driving further increases in body size.

In conclusion, it is clear that desiccation resistance in *L. humile* is a phenotype influenced by a variety of different potential drivers. Here, we show a clear role for body size, which is correlated with supercolony identity in California populations. In addition, our analysis of CHC profiles identified specific CHCs that were both positively and negatively correlated with desiccation resistance, but no overarching patterns relating particular CHC classes or overall amounts of CHCs with desiccation resistance. Interestingly, workers from the large supercolony that dominates most of the introduced range in California displayed both the largest body sizes and the greatest levels of desiccation resistance, suggesting that future studies should explore the potential link between body size, desiccation resistance and invasion success. Finally, while more research would be needed to increase confidence in this pattern, currently it appears that the CHC differences between invasive supercolonies represent a scale of variation that is enough to maintain colony boundaries, but not enough to explain differences in desiccation resistance, which might mean socially relevant CHC information at this scale can vary without inducing a functional trade-off with cuticular waterproofing.

## Supplementary Material

10.1242/jexbio.245578_sup1 Supplementary information
