# Supplementary material for: The role of body size and cuticular hydrocarbons in the desiccation resistance of invasive Argentine ants (Linepithema humile)
Source: J Exp Biol. 2023 Aug 21;226(16):jeb245578. doi: 10.1242/jeb.245578 (PMC10482004; doi:10.1242/jeb.245578)
Supplement: Supplementary information [file jexbio-226-245578-s1.pdf]

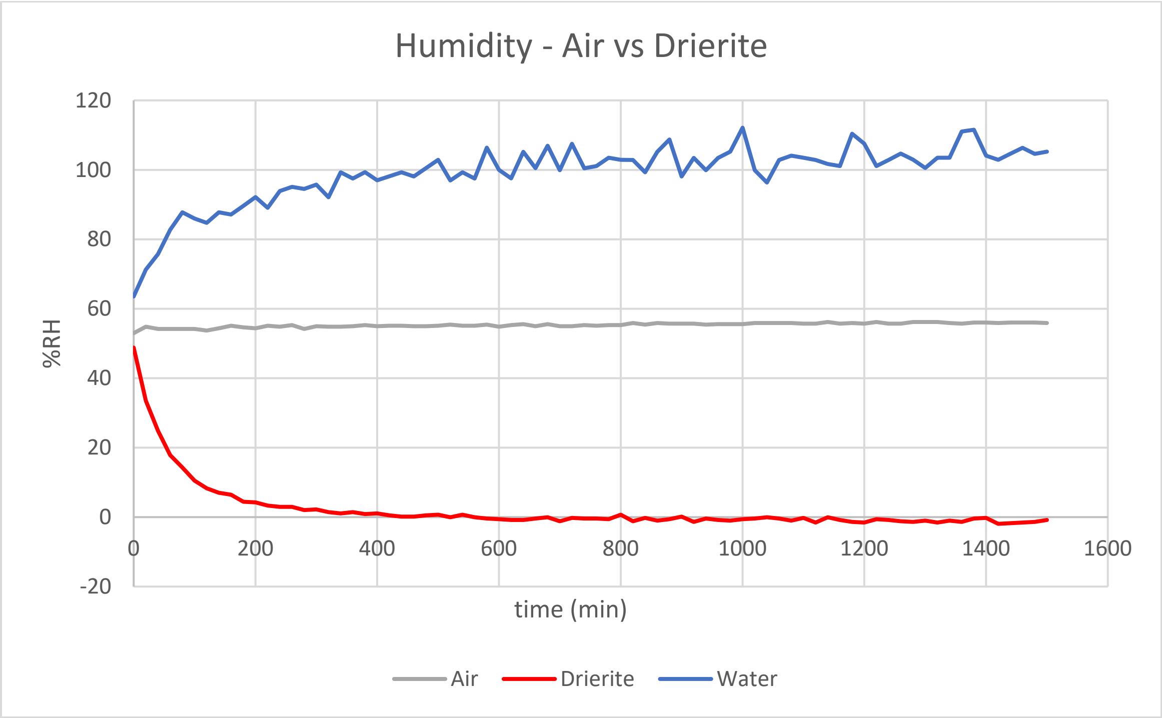

Fig. S1. Relative humidity of assay tubes over time. Humidity was recorded with iButton Hygrochron sensors (iButtonLink©, Innovation Drive Whitewater, WI, United States). These sensors could not fit into the 15 ml conical tubes used in experiment, so we placed iButtons into 50 ml conical tubes on top of two cotton balls with our air, drierite, or water treatment below (see Figure 2 in the manuscript).

Journal of Experimental Biology • Supplementary information

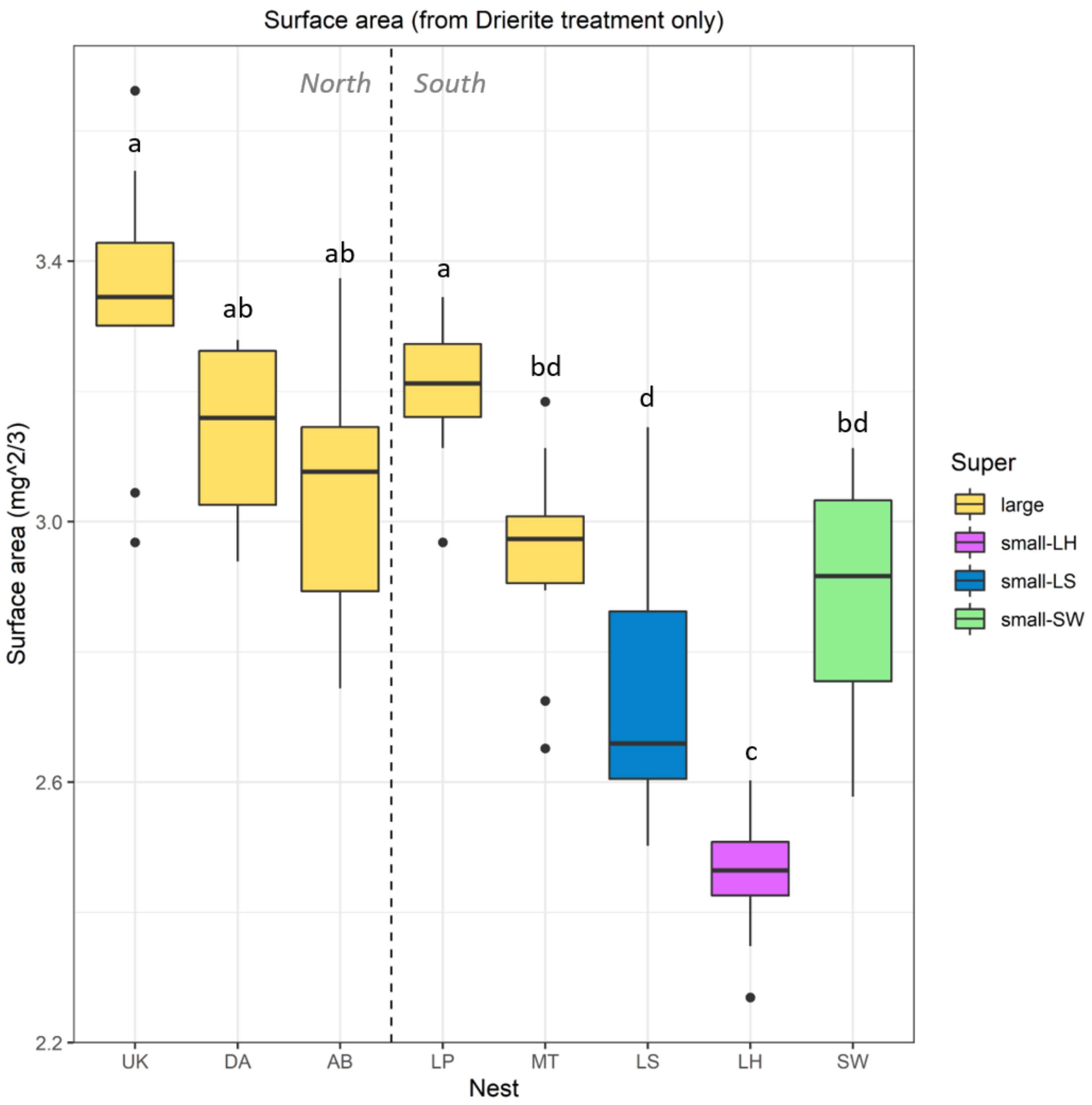

Fig. S2. Surface area box plots for each nest. Surface area is estimated from body mass, and body mass was measured for each replicate group of ants in the Drierite treatment (i.e., severe desiccation treatment). Colors indicate supercolony identity. The vertical dotted line indicates nests from northern vs. southern California. Nest abbreviations are explained in the main manuscript. Significant differences between the nests were tested with a pairwise permutations analysis of the variance (full results of these ANOVA tests are in Supplemental Data 2).

Journal of Experimental Biology • Supplementary information

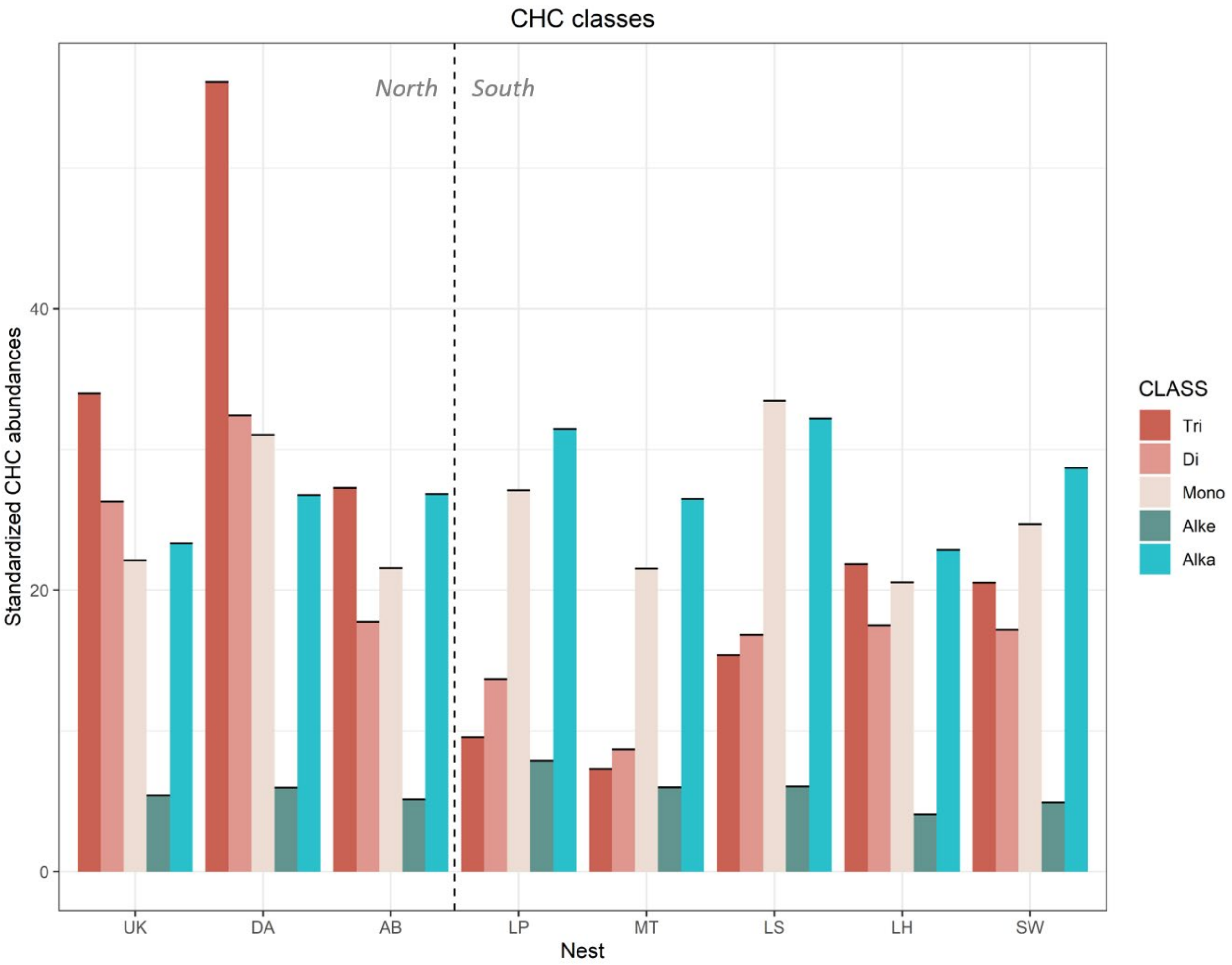

Fig. S3. CHC classes using standardize abundances. Each bar is the sum of standardized CHC abundances for all compounds in a class, standardized against one candidate Argentine ant CHC profile. These standardized abundances are nest-specific and are not weighted by body mass or surface area of the experimental subjects, so there are no error bars in this graph. The five color classes are *n*-alkanes ("Alka"), *n*-alkenes ("Alke"), and methyl-branched alkanes with one ("Mono"), two ("Di") or three ("Tri") methyl branches. The nest abbreviations are explained in the methods section of the manuscript.

Journal of Experimental Biology • Supplementary information

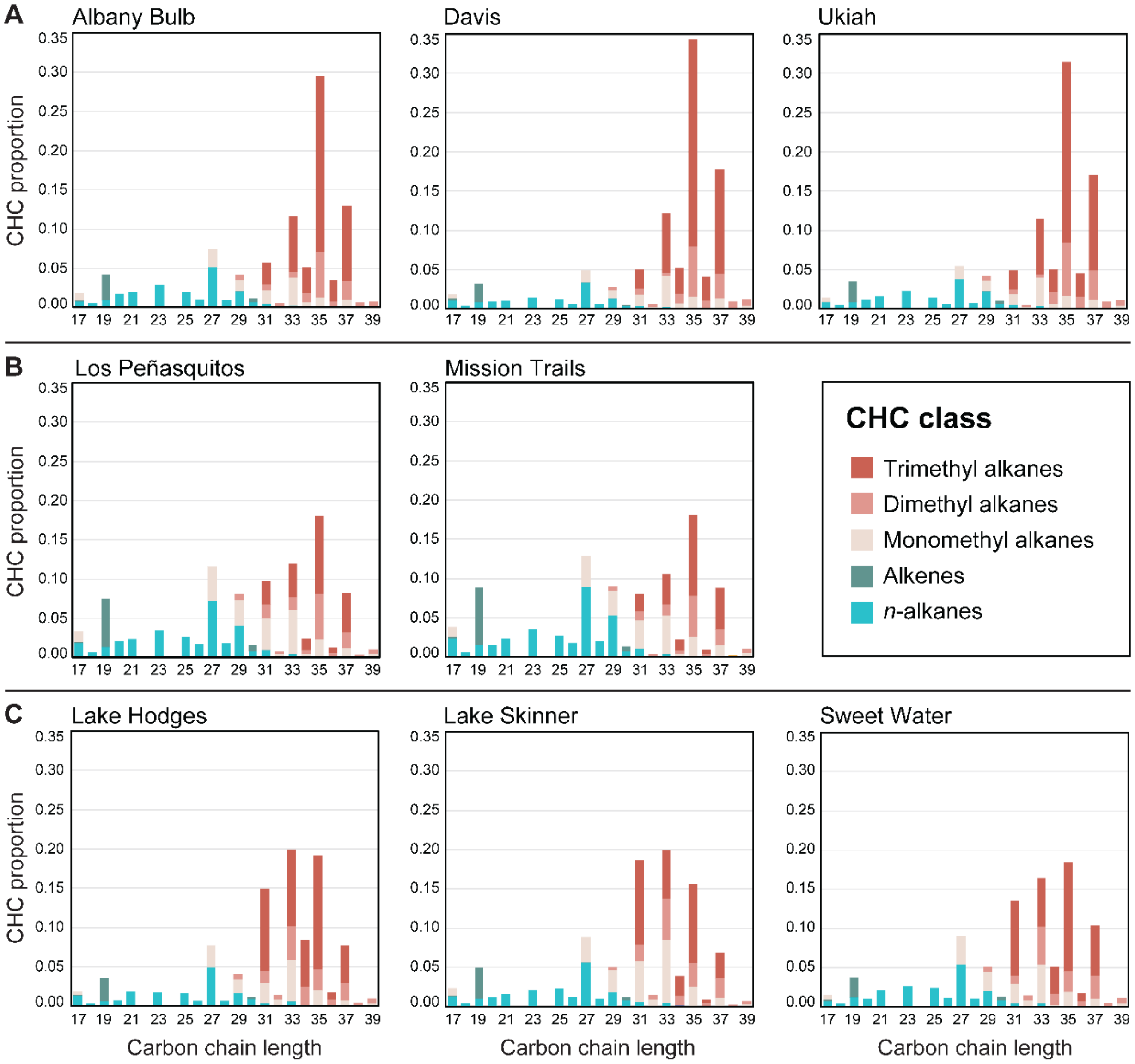

Fig. S4. Complete CHC profile proportions for each nest. Stacked bar plots of *L. humile* CHC proportions including all CHCs detected (i.e., *n*-alkanes, methyl branched alkanes, and *n*-alkenes), with methyl-branched alkanes divided into subcategories based on their number of methyl branches (i.e., one, two, or three). CHC proportions are organized by carbon chain length on the x-axis and CHC profiles are grouped according to supercolony identity and location: (A & B) large supercolony sites, (C) small supercolonies, (A) northern California, and (B & C) southern California.

Journal of Experimental Biology • Supplementary information

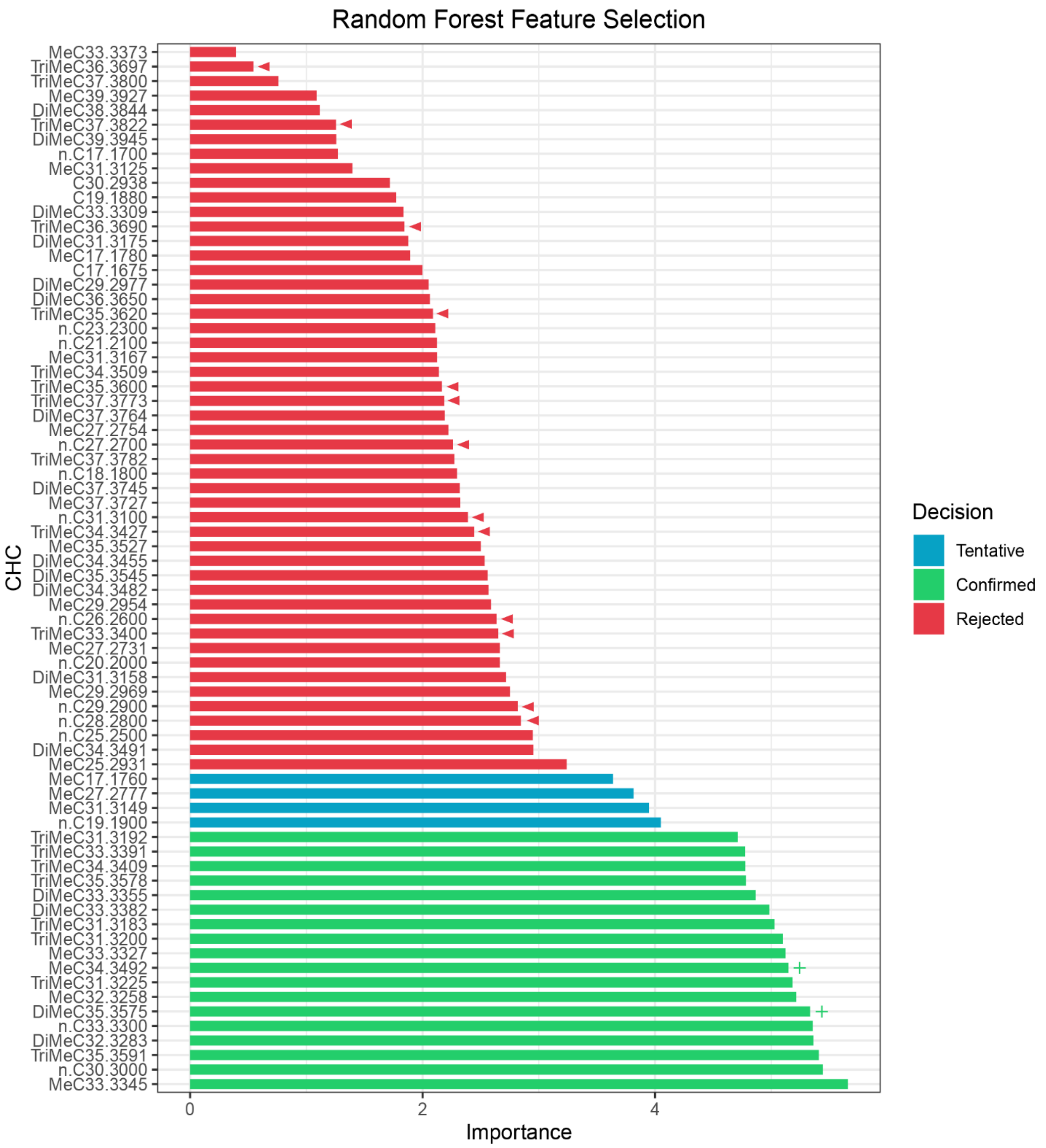

Fig. S5. Random Forest feature selection with standardized CHCs. The features are all of the recorded cuticular hydrocarbons (CHCs) found from our nest collections, used in random forest decision trees to predict LT50 (from the drierite treatment, specifically). This is an alternative version of Figure 5 in the manuscript, using standardized CHC abundances instead of the CHC mass per body surface (ng/mg^$^{2/3}$) data. Spearman correlation results for each CHC can be found in Supplemental Data 4.

Journal of Experimental Biology • Supplementary information
